# The Modern Practice of Midwifery

**Published:** 1859-04

**Authors:** 


ART. VI.—*The Modern Practice of Midwifery. A course of Lectures on Obstetrics: delivered at the St. Mary's Hospital, London.* By WM. TYLER SMITH, M. D., Member of the Royal College of Physicians. With an Introductory Lecture on the History of the Art of Midwifery, and Copious Practical Annotations, by AUGUSTUS K. GARDNER, A. M., M. D., late Instructor on Obstetrics in the N. Y., Preparatory School of Medicine. Author of the " Causes and Curative Treatment of Sterility," etc. Illustrated by 212 engravings. New York: ROBERT M. DE WITT, Publisher, 160 and 162 Nassau street.

The work before us is a republication from the pages of the *London Lancet*, of the lectures of Dr. Smith. These lectures have been already embodied in a systematic work on Midwifery published in London; but here they have been preserved in their original form, prcceeded by an introductory lecture on the history of Midwifery delivered by Dr. Gardner, in 1851, at the N. Y. College of Physicians and Surgeons, to which are added copious annotations by the American editor.

In the editor's preface we find the hope expressed, that the work as now issued, "may not fall under the general obloquy of American editions of foreign works." We would enquire, what is the cause of the general, or, we would prefer, the frequent objections of the profession of this country to foreign republications? Certainly if works issued on the other side of the Atlantic are of sufficient value to warrant their republication here, we should be obliged to our publishers for giving them to us at a smaller cost than the foreign editions, with a saving of the expense of duty, transportation, etc! But what is objectionable in these republications, is the quantity of editorial notes and comments, and the well worn illustrations, by which they are almost always adorned, diverting from the appropriate channels of our national literature the original labors of our distinguished men; for then, American researches appear tacked on, as it were, to the work of some European celebrity, instead of helping to build up a national literature.

This is an injustice which is done to our own eminent men; but, on the other hand, there is frequently an injustice to the foreign authors, of which they might reasonably complain. Their books are edited by men who only wish to link their names with that of a distinguished author, by means of notes, etc., which only encumber the text, and frequently, by their puerility, injure the character of the original work; one can always be successful if he look after deficiencies with the desire to find them, in order to increase his own importance. We do not approve of these copious annotations of American editors; if Enropean writers ignore our advances in science, let them be rebuked through the pages of our Journals, some of which are extensively circulated in other countries, and let us have the pages of their own works as they originally appeared. There are valuable American works in the several departments of our science, which, taking cognizance of *European* labor, are the proper exponents of *American* advancement.

The work before us is valuable, and worthy of the high reputation of its distinguished author, but we should have been mnch better satisfied if it had appeared without the introductory lecture of Dr. Gardner, his paper on "obstructed labor from rigidity of the os uteri," which was read before the N. Y. Academy, and his copious annotations. These two papers by Dr. Gardner are introduced with no apology, either on the part of the author, or implied by a peculiar pertinence. The zeal of the editor has also led him to embellish a page with the horrid wood cut representing a female figure placing another in a proper position for the use of the "elevator perinei," which appeared in Dr. J. Marion Sims' tawdry address before the New York Academy of Medicine.

We cannot enter in a review of Dr. Smith's woik, and can only state, from the examination which we have made of it, our good opinion of it as a practical work on midwifery. It is hardly as complete, however, as many text-books which we have on this subject, and in all probability, not so much adapted to this end as the work which appeared in London.

